# Minimum standards for physical therapists providing rehabilitation care for people with chronic respiratory diseases in Japan: An e-Delphi study

**DOI:** 10.1371/journal.pone.0344464

**Published:** 2026-03-10

**Authors:** Kosuke Sasaki, Manabu Sezaki, Kiyokazu Sekikawa, Hiroshi Kiga, Madoka Iwasaki, Kazuyuki Tabira, Yutaro Oki, Akira Tamaki

**Affiliations:** 1 Department of Rehabilitation, Kobe City Medical Center General Hospital, Kobe, Hyogo, Japan; 2 Physical Therapy Standardization Committee, Japanese Society of Respiratory Physical Therapy, Tokyo, Japan; 3 Department of Rehabilitation, Saiseikai Niigata Kenoh Kikan Hospital, Sanjo, Niigata, Japan; 4 Department of Physical Analysis and Therapeutic Sciences, Graduate School of Biomedical and Health Sciences, Hiroshima University, Hiroshima, Japan; 5 Department of Rehabilitation, Niigata Prefectural Central Hospital, Joetsu, Niigata, Japan; 6 Department of Clinical Technology, Niigata University Medical and Dental Hospital, Niigata, Japan; 7 Graduate School of Health Science, Kio University, Kitakatsuragigun-koryocho, Nara, Japan; 8 Kobe University Graduate School of Health Sciences, Kobe, Hyogo, Japan; 9 Department of Physical Therapy, School of Rehabilitation, Hyogo Medical University, Kobe, Hyogo, Japan; La Trobe University - Bundoora Campus: La Trobe University, AUSTRALIA

## Abstract

Rehabilitation interventions, including structured pulmonary rehabilitation (PR) programs, can improve symptoms, exercise tolerance, and quality of life for people with chronic respiratory diseases. However, in Japan, physical therapists provide rehabilitation care across care pathways both within and outside formal PR programs, and consensus-based minimum standards for physical therapists’ essential knowledge and skills have not yet been clearly articulated. This study aimed to establish expert consensus on the minimum standards (essential knowledge and skills) required for physical therapists providing rehabilitation care for people with chronic respiratory diseases in Japan, irrespective of whether care is delivered within a formal pulmonary rehabilitation program. A two-round e-Delphi study was conducted. An expert panel of 39 experienced physical therapists from Japanese clinical and academic settings evaluated 114 potential items for inclusion as minimum standards based on a predefined 70% consensus threshold. Consensus was achieved for 105 items. The final standards span knowledge relevant to chronic respiratory disease (CRD) rehabilitation care (including common management approaches and relevant devices), patient assessment and outcome measurement (including respiratory and physical function, activity/activities of daily living (ADL), and patient-reported outcomes), and rehabilitation interventions (including exercise and activity/ADL training). Items specific to invasive line management/monitoring (e.g., arterial line–related competencies) did not reach consensus, consistent with the intent to define baseline standards across CRD rehabilitation contexts rather than intensive care unit (ICU)-specific practice standards. These standards could inform training curricula and continuing professional development, help guide policy-making and service planning by clarifying baseline expectations across care settings, and provide a reference point for future cross-national comparisons following contextual adaptation. Future research should evaluate implementation feasibility in diverse healthcare settings and support periodic review and updating of the standards as clinical evidence and service contexts evolve.

## Introduction

The prevalence of chronic respiratory diseases (CRDs) was estimated at 454.6 million cases in 2019 [1], a figure expected to rise with global population growth and aging [2]. CRDs were the third leading cause of death worldwide in 2019 [1]. These diseases impose a high burden [1], with approximately 50% of people experiencing difficulties in activities of daily living (ADLs) [3,4] and diminished quality of life (QOL) [5–7].

Pulmonary rehabilitation (PR) is a structured, multidisciplinary program that combines patient education, exercise training, nutritional support, and psychosocial support, and has been shown to improve dyspnea, exercise tolerance, and QOL in people with CRDs [8–12]. International clinical practice guidelines and policy statements consistently recommend PR and emphasize exercise-based rehabilitation, with physical therapists playing a key role in assessment, individualized exercise prescription, and symptom and functional monitoring [13–16]. However, physical therapy is not synonymous with PR, and physical therapists may provide rehabilitation care for people with CRDs both within and outside formal PR programs across care pathways (e.g., in primary-care physical therapy practice for chronic obstructive pulmonary disease) [17,18].

Despite the established effectiveness of PR, access to and uptake of PR remain inconsistent across countries and healthcare systems, including substantial variation in service availability, referral, and participation [15,19–23]. Barriers to delivering and accessing PR (and rehabilitation care for people with CRDs more broadly), including referral to PR where available, are multifactorial and operate at multiple levels including system/service factors, clinician factors, and patient-level access/participation constraints, —beyond clinicians’ knowledge alone—encompassing service capacity and resources, variability in referral pathways and interprofessional coordination, and clinicians’ knowledge, beliefs, and environmental constraints [15,16,18,24–26]. Such implementation gaps, together with limited standardization of assessment and outcome measurement in CRD rehabilitation, may contribute to heterogeneity in rehabilitation content and evaluation across care settings [27–30].

In response to these implementation and standardization challenges, international initiatives have sought to harmonize respiratory physical therapy education and competency expectations, exemplified by the European Respiratory Society Respiratory Physiotherapy HERMES syllabus and harmonised curriculum [31–33]. International clinical practice guidelines and policy statements for pulmonary rehabilitation also define core program components and describe the role of physical therapists within structured PR [13–16]; however, they generally do not provide consensus-based minimum standards that apply to physical therapists delivering rehabilitation care for people with CRDs across the broader care continuum (including settings outside formal PR programs). In parallel, consensus-based minimum standards for physiotherapists have been developed internationally in other specialized settings (e.g., critical care), including Australia/New Zealand, the United Kingdom, South Africa, Nigeria, and Japan, demonstrating the feasibility of Delphi-derived frameworks to articulate baseline expectations in physical therapy practice [34–38]. Nevertheless, published consensus-based minimum standards specifically targeting physical therapists providing rehabilitation care for people with CRDs across care settings remain limited, despite growing international work on outcome domains, core outcome sets, and related educational content in pulmonary rehabilitation and CRD care [17,28,39–41].

This gap is particularly relevant in Japan, where the rapid growth in the physical therapist workforce has heightened the need for a structured educational system to ensure consistent, high-quality rehabilitation care for people with CRDs, especially among newly qualified therapists [42,43]. Drawing on literature on applicability, transferability, and adaptation in health guidance development and implementation [44,45], the applicability and transferability of minimum standards may be influenced by contextual differences (e.g., healthcare system organization and resources, educational pathways and scope of practice, and cultural expectations regarding professional roles and patient engagement). Accordingly, we present the current minimum standards as a Japan-specific baseline; when considering use in other settings, local stakeholders should assess relevance and feasibility and adapt as needed. Establishing consensus-based minimum standards could support clinical practice and education by providing a shared baseline for essential knowledge and skills. Therefore, this study aimed to establish expert consensus on the minimum standards (knowledge and skills) required of physical therapists providing rehabilitation care for people with CRDs in Japan, irrespective of whether care is delivered within a formal PR program.

## Materials and methods

### Study design, data source, and ethical considerations

This study was designed and reported in accordance with the CREDES (Guideline for Conducting and REporting DElphi Studies) guidelines [46]. A completed CREDES checklist is provided as a supplementary file (S1 Table). In this study, we used the electronic-Delphi (e-Delphi) method with two rounds of voting to establish a consensus among physical therapists on minimum standards (knowledge and skills) for physical therapists providing rehabilitation care for people with CRDs. The Delphi method is a technique used to achieve consensus among a panel of experts on a specific issue [47]. The e-Delphi method, a web-based variant, integrates the expertise of geographically dispersed groups and allows for rapid feedback between experts and researchers [48].

This study was approved by the Ethical Review Committee of Niigata Prefectural Central Hospital (approval number: 2304). All participants were informed about the study's purpose, procedures, and their rights to withdraw. Written informed consent was obtained electronically using Google Forms (Google LLC, CA, USA) prior to their participation.

### Expert panel

Although there is no universally accepted standard for the size of an expert panel, 30–50 members is considered appropriate [49]. Therefore, this study targeted approximately 40 expert panel members. To recruit physical therapists with adequate knowledge and experience in respiratory physical therapy for people with CRDs, a purposive and snowball sampling approach was utilized [49]. Initially, 45 officers of the Japanese Society of Respiratory Physical Therapy (JSRPT) were included as potential candidates. Additionally, the researchers (M.S., Ki.S., and K.T.) identified 6 candidates from among the professional JSRPT members.

The criteria for inclusion in the expert panel were as follows:

- Membership as a Specialist Member A (Japanese: Senmon-kaiin A) of JSRPT. Specialist Member A is a JSRPT membership category indicating advanced expertise, based on predefined eligibility criteria published by the society [50].
- At least five years of experience treating people with CRDs in a medical facility.
- Holding at least one credential indicating formal training in respiratory physical therapy and/or respiratory care: a Japanese Physical Therapy Association credential—Certified Physical Therapist (Pulmonary disease) and/or Specialist Physical Therapist (Respiratory Physical Therapy / Cardiovascular Physical Therapy / Diabetes Physical Therapy) [51]; (Three-Society Joint) Certified Respiratory Therapist (Japanese: 3-gakkai godo kokyu ryoho nintei-shi) [52]; or Respiratory Care Instructor (Japanese: Kokyu Care Shidoshi) [53].
- If the candidate had not treated people with CRDs in a clinical setting within the past two years, evidence of current professional engagement in respiratory physical therapy was required, defined as either (i) authorship of at least one peer‑reviewed publication on respiratory physical therapy within the past two years, or (ii) teaching a university-level course that includes respiratory physical therapy content (e.g., cardiorespiratory/respiratory physical therapy) within the past two years.

This criterion was included to apply a pragmatic recency threshold for domain expertise among panelists without recent clinical practice.

All panelists were licensed physical therapists; the credentials and professional activities listed above were prespecified indicators of expertise in respiratory physical therapy and/or respiratory care. Because the aim of this study was to establish minimum standards (knowledge and skills) for physical therapists providing rehabilitation care for people with CRDs, eligibility was intentionally restricted to licensed physical therapists with advanced expertise in respiratory physical therapy as operationalised by the prespecified JSRPT criteria.

Between June 25, 2023, and June 30, 2023, invitations to join the expert panel were sent by email. The invitations detailed the study's purpose and informed the candidates of their right to withdraw from the study. Candidates who agreed to participate were considered for inclusion in the study. No financial or other forms of compensation were offered for participation.

Data on the following characteristics of the respondents were collected: age, years of experience as a physical therapist, highest educational qualification, primary work setting, the number of peer-reviewed publications on respiratory physical therapy, and the presence of any respiratory-related professional credentials (respiratory physical therapy and/or respiratory care; multiple responses allowed).

### Development of the questionnaire

Members of the JSRPT Standardization Committee developed the initial pool of candidate minimum standards through a targeted review of key documents selected a priori to ensure coverage of essential domains and relevance to the Japanese clinical context (rather than a systematic/scoping review). Primary Japan‑specific sources included the national clinical practice guideline for physical therapy in respiratory disorders [54], a Japan-specific review compiling outcome measures used/validated in respiratory physical therapy [55], and a national Japanese statement on respiratory rehabilitation [56]. To incorporate an international perspective on pulmonary rehabilitation content and outcome domains, the committee also consulted an official American Thoracic Society workshop report defining modern pulmonary rehabilitation [18]. In addition, an international consensus paper was screened to confirm coverage of patient education and self‑management domains; details of this supplementary screening source and its role are provided in S1 Appendix (Table A). Finally, to inform respiratory‑related acute‑care concepts that may be encountered across care pathways, the committee consulted a Japanese minimum‑standards/Delphi study for physical therapists working in intensive care [38] and extracted only respiratory‑related concepts within the scope of this study (including basic mechanical ventilation concepts); intensive care unit (ICU)‑specific devices/monitoring and non‑respiratory critical‑care conditions were treated as out of scope (see S1 Appendix, Section A). Three committee members (M.S., H.K., and M.I.) extracted candidate concepts and drafted the questionnaire items in Japanese, which were reviewed by three additional committee members (Ko.S., Ki.S., and K.T.) and refined using prespecified rules (e.g., duplicate removal, merging/splitting where appropriate, wording clarification/terminology harmonization, and scope specification) to reduce redundancy and improve clarity. Based on these sources and iterative committee review, the final candidate minimum standards comprised 114 items. The questionnaire was created using Google Forms and was internally pretested by six committee members to confirm comprehensibility and technical functionality, leading to minor wording revisions; a separate external pilot with non‑panelists was not conducted. Further details, including representative examples and an item‑level audit trail of pre‑Delphi amendments, are provided in S1 Appendix (Table B) and S2 Table.

### e-Delphi round

Because there is no universally accepted standard threshold for defining consensus in Delphi studies [57–59], consensus criteria should be specified a priori to enhance transparency and reproducibility [57,58]. We therefore prespecified consensus for inclusion as a supermajority criterion: ≥ 70% of panelists rating an item as 4 (“recommended”) or 5 (“highly recommended”) on the five-point Likert scale. We selected this cutoff to balance inclusiveness and stringency when identifying minimum standards for use across diverse rehabilitation contexts for chronic respiratory disease, and it is consistent with thresholds used in Delphi studies that develop minimum standards in specialized physical therapy practice settings (e.g., critical care) [34,36–38]. Methodological reviews further indicate that consensus is commonly operationalised using percentage agreement, although the specific threshold varies across studies [57–59].

The e-Delphi rounds were conducted anonymously. In the first round, the participants were asked to evaluate 114 items and determine whether each item should be included as a minimum standard. A five-point Likert scale was used to score each item, with respondents selecting from the following options: 1 = not at all recommended, 2 = not recommended, 3 = neutral, 4 = recommended, and 5 = highly recommended. Items for which ≥70% of the respondents in that round selected 4 (“recommended”) or 5 (“highly recommended”) were considered to have achieved consensus for inclusion as minimum standards [34,36–38,57–59]. Among these items, those with a median score of 5 were classified as “highly recommended,” and those with a median score of 4 were classified as “recommended.” Items for which consensus was not reached in the first round were carried over to the second round. Items that did not meet the ≥ 70% inclusion criterion after Round 2 were designated as “no consensus”. The response period for the first round was from July 3, 2023, to July 21, 2023.

In the second round, participants were again asked to evaluate the items for which consensus had not been reached in the first round using the same criteria. The respondents were provided with the results of the first round, including the consensus level, median, and interquartile range for each item. The second round was conducted from July 31, 2023, to August 18, 2023.

### Statistical analysis

The data were exported to Microsoft Excel 2021 (Microsoft Corporation, WA, USA) for analysis. The characteristics of the study participants and the consensus levels for the minimum standards are presented as numbers and percentages. The median and interquartile range for each item are also provided.

## Results

Of the 51 expert panel candidates, 44 agreed to participate in the study. Among these participants, 39 (89%) participated in both the first and second rounds of the Delphi survey.

The 39 participants who completed both rounds represented a highly experienced group of physical therapists. The majority reported over 10 years of clinical experience, held respiratory-related professional credentials (e.g., certifications/specialist credentials in respiratory physical therapy and/or respiratory care), and reported peer-reviewed publications on respiratory physical therapy. The panel was evenly balanced between experts from medical facilities (51%) and those from academic or educational institutions (49%) (Table 1).

**Table 1:** Characteristics of the participants (n = 39).

Characteristics		
Age group, n (%)		
20–29 years old	0	(0)
30–39 years old	10	(26)
40–49 years old	17	(44)
50–59 years old	11	(28)
60 years or older	1	(3)
Clinical experience as a physical therapist, n (%)		
< 10 years	1	(3)
10–19 years	16	(41)
20–29 years	13	(33)
30–39 years	9	(23)
Type of work facility, n (%)		
Medical facility	20	(51)
Long-term care facility	0	(0)
University/educational institution	19	(49)
Other	0	(0)
Peer-reviewed publications on respiratory physical therapy, n (%)		
0 publications	3	(8)
1–4 publications	14	(36)
≥5 publications	22	(56)
Professional credentials (multiple responses allowed ^a^), n (%)		
Certified Physical Therapist (Pulmonary disease)	22	(56)
Specialist Physical Therapist (Respiratory Physical Therapy / Cardiovascular Physical Therapy / Diabetes Physical Therapy)	26	(67)
(Three-Society Joint) Certified Respiratory Therapist	29	(74)
Respiratory Care Instructor (Kokyu Care Shidoshi)	22	(56)
Highest qualification, n (%)		
Diploma	9	(23)
Bachelor's degree	8	(21)
Master's degree	7	(18)
Doctoral degree	15	(38)

^a^For professional credentials, multiple responses were allowed; percentages are calculated using the total number of participants who completed both rounds (n = 39) as the denominator.

**Fig 1:**
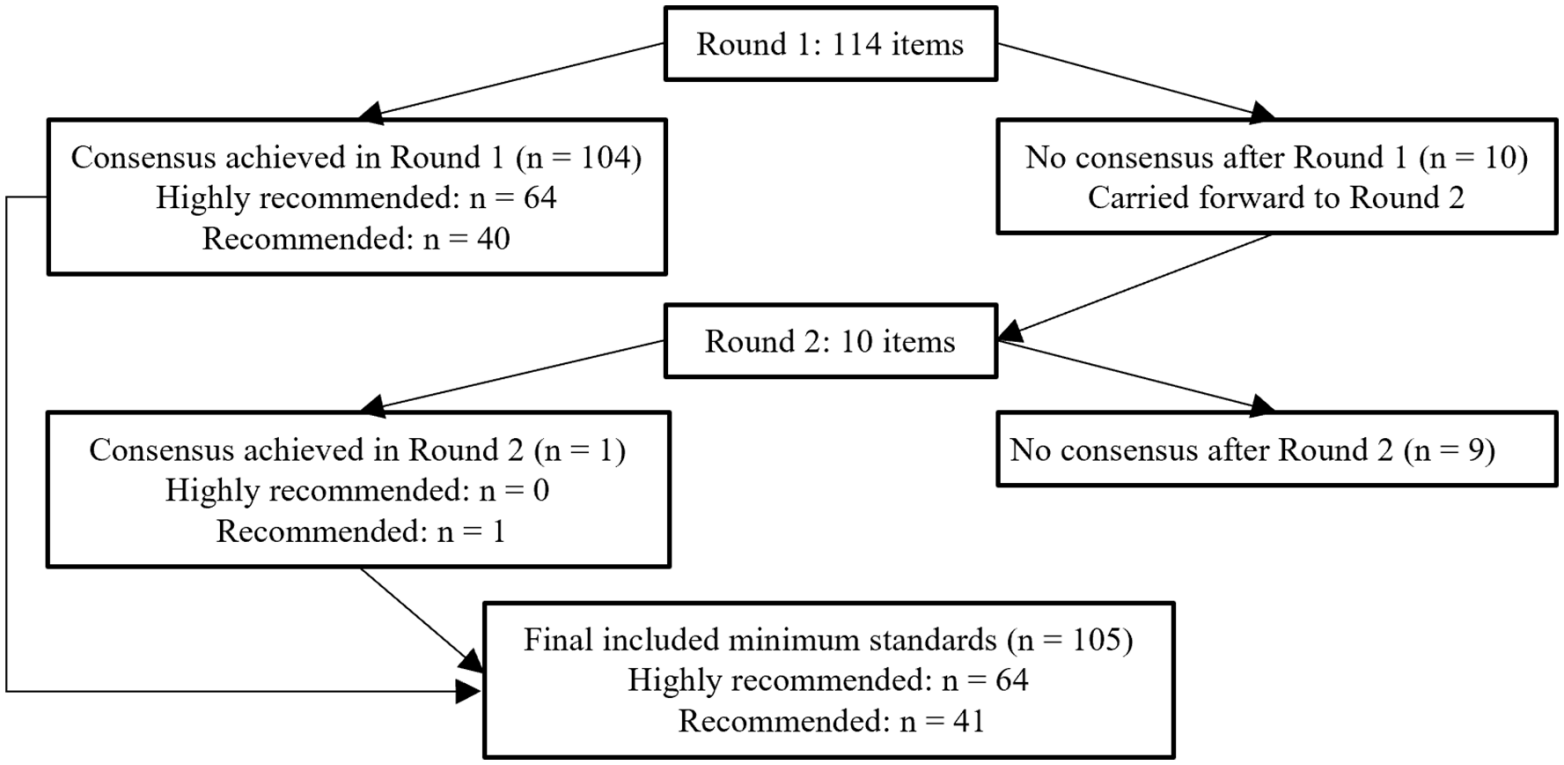
The flow of rounds throughout the e-Delphi study and summary of consensus outcomes. In Round 1, consensus for inclusion (≥70% of panelists rating an item as 4 [recommended] or 5 [highly recommended]) was achieved for 104 of 114 items, and 10 items were carried forward to Round 2. In Round 2, one additional item met the inclusion criterion, resulting in 105 items included as minimum standards (64 classified as “highly recommended” and 41 as “recommended”) and nine items with no consensus. Items are organized by domain/category in Table 2 and S4 Table, and a domain-level summary of final designations is provided in S5 Table to enable rapid identification of areas with stronger consensus (items classified as “highly recommended”).

In the first round, ≥ 70% of the respondents rated 104 of the 114 items as “highly recommended” or “recommended” for inclusion as minimum standards, whereas a consensus was not reached for ten items. In the second round, one of the ten items for which a consensus was not achieved in the first round was rated as “highly recommended” or “recommended” by ≥ 70% of the respondents (Table 2). Ultimately, consensus was achieved on 105 items for inclusion as minimum standards through the Delphi rounds. Of these items, 64 were classified as “highly recommended,” and 41 items were classified as “recommended.” A consensus was not reached for nine items (Fig 1). To facilitate rapid identification of areas with stronger consensus, items are organized into seven clearly labeled domains/categories (Domains 1–7) in Table 2 and S4 Table (knowledge of CRDs, medical devices/equipment, mechanical ventilation, pathophysiology, medical tests, assessment items, and programs), and within each domain/category items classified as “highly recommended” (stronger consensus) versus “recommended” are explicitly indicated in the tables. Of the 105 items included as minimum standards, the largest number related to assessment items (n = 39), whereas no-consensus items were concentrated in the medical devices/equipment domain (n = 4) and the assessment domain (n = 3) (S4 Table). Detailed round-by-round results for all 114 items are available in S3 Table, and the final consensus designation for each candidate item (n = 114)—“highly recommended,” “recommended,” or “no consensus”—is presented in S4 Table. A domain-level summary of the final consensus designations is provided in S5 Table. Balance- and falls-related constructs were not included as prespecified candidate items and therefore were not evaluated during the Delphi rounds; accordingly, they are not represented as standalone items in Table 2 and S3–S4 Tables.

**Table 2:** Round-by-round agreement and final consensus designation for each candidate item (n = 114), organized by domain/category (Domains 1–7).

Domain/category and items	Round 1: “Highly Recommended” or “Recommended” (%)	Round 2: “Highly Recommended” or “Recommended” (%)	Final designation	Notes
**Domain 1. Possessing Knowledge on the Following Chronic Respiratory Diseases:**				
Guidelines on Chronic Respiratory Diseases	100	NA	Highly recommended	
Guidelines on Respiratory Physical Therapy for Chronic Respiratory Diseases	97.4	NA	Highly recommended	
Bronchodilators	97.4	NA	Recommended	
Inhalation Devices	79.5	NA	Recommended	
Steroids	87.2	NA	Recommended	
Antibiotics	64.1	74.4	Recommended	Re-rated in Round 2
Acute Exacerbations	100	NA	Highly recommended	
Smoking Cessation	92.3	NA	Highly recommended	
Social Resources and Welfare	87.2	NA	Recommended	
Long-Term Care Insurance	79.5	NA	Recommended	
Self-Management	100	NA	Highly recommended	
Advance Care Planning	87.2	NA	Recommended	
Multidisciplinary Interventions and Respiratory Care Support Teams	92.3	NA	Highly recommended	
Home Oxygen Therapy	100	NA	Highly recommended	
Home Mechanical Ventilation	89.7	NA	Highly recommended	
**Domain 2. Understanding and Interpreting Various Medical Devices and Equipment:**				
Oxygen therapy: Low-flow system	100	NA	Highly recommended	
Oxygen therapy: High-flow system	94.9	NA	Highly recommended	
Oxygen therapy: High-flow nasal cannula therapy	94.9	NA	Highly recommended	
Oxygen therapy: Home oxygen therapy	100	NA	Highly recommended	
Oxygen therapy: Home oxygen therapy (liquid oxygen)	92.3	NA	Recommended	
Arterial Line	56.4	61.5	No consensus	Re-rated in Round 2
Central Venous Catheter	56.4	64.1	No consensus	Re-rated in Round 2
Tracheostomy, Tracheal Cannula, Minitracheostomy	87.2	NA	Recommended	
Indwelling Urinary Catheter	43.6	51.3	No consensus	Re-rated in Round 2
Chest Drain	82.1	NA	Recommended	
Nasogastric Tube	56.4	64.1	No consensus	Re-rated in Round 2
Suction Device	94.9	NA	Highly recommended	
Cough Assist Device	84.6	NA	Recommended	
Oxygen Cylinder	100	NA	Highly recommended	
**Domain 3. Understanding and Interpreting Mechanical Ventilation:**				
Intubated Mechanical Ventilation	97.4	NA	Highly recommended	
Noninvasive Ventilation	100	NA	Highly recommended	
Synchronized Intermittent Mandatory Ventilation	87.2	NA	Recommended	
Volume-Controlled Ventilation	94.9	NA	Recommended	
Pressure-Controlled Ventilation	94.9	NA	Recommended	
Positive End-Expiratory Pressure	97.4	NA	Recommended	
Pressure Support	97.4	NA	Highly recommended	
Continuous Positive Airway Pressure	97.4	NA	Highly recommended	
Adaptive Servo-Ventilation	71.8	NA	Recommended	
Protocols for Weaning from Mechanical Ventilation	94.9	NA	Highly recommended	
**Domain 4. Understanding and Interpreting Pathophysiology:**				
Chronic Obstructive Pulmonary Disease	100	NA	Highly recommended	
Interstitial Lung Disease	100	NA	Highly recommended	
Pulmonary Tuberculosis/Post-Tuberculosis Sequelae	89.7	NA	Recommended	
Bronchial Asthma	97.4	NA	Highly recommended	
Nontuberculous Mycobacterial Infections	89.7	NA	Recommended	
Lung Cancer	100	NA	Highly recommended	
Pulmonary Hypertension	97.4	NA	Recommended	
Scoliosis	66.7	46.2	No consensus	Re-rated in Round 2
Pneumonia	100	NA	Highly recommended	
Pneumothorax	94.9	NA	Recommended	
Pleural Effusion	94.9	NA	Recommended	
Frailty	97.4	NA	Highly recommended	
Sarcopenia	97.4	NA	Highly recommended	
**Domain 5. Understanding and Interpreting the Results of Various Medical Tests:**				
Pulmonary Function Test (FEV, VC, etc.)	100	NA	Highly recommended	
Pulmonary Function Test (DLCO)	100	NA	Highly recommended	
Chest X-ray	100	NA	Highly recommended	
Chest CT	97.4	NA	Highly recommended	
Chest MRI	71.8	NA	Recommended	
Electrocardiogram	92.3	NA	Recommended	
Cardiac Ultrasound	82.1	NA	Recommended	
Blood Test: Hematology	92.3	NA	Highly recommended	
Blood Test: Biochemistry	94.9	NA	Highly recommended	
Sputum Culture	61.5	48.7	No consensus	Re-rated in Round 2
Pulse Oximetry (SpO2)	100	NA	Highly recommended	
End-Tidal CO2 Pressure (PETCO2)	82.1	NA	Recommended	
Arterial Blood Gas Analysis	100	NA	Highly recommended	
Nutritional Status Assessment (BMI, %IBW, %LBW, etc.)	100	NA	Highly recommended	
**Domain 6. Able to Perform and Interpret the Following Assessment Items:**				
Japan Coma Scale	84.6	NA	Highly recommended	
Glasgow Coma Scale	82.1	NA	Highly recommended	
Richmond Agitation-Sedation Scale	82.1	NA	Highly recommended	
Physical Assessment of the Respiratory System: Inspection	100	NA	Highly recommended	
Physical Assessment of the Respiratory System: Palpation	100	NA	Highly recommended	
Physical Assessment of the Respiratory System: Auscultation	100	NA	Highly recommended	
Physical Assessment of the Respiratory System: Percussion	84.6	NA	Highly recommended	
Respiratory Rate	100	NA	Highly recommended	
Evaluation of the Breathing Pattern	100	NA	Highly recommended	
Cough Ability (Cough Peak Flow)	92.3	NA	Highly recommended	
Joint Range of Motion	79.5	NA	Recommended	
Respiratory Muscle Strength (PEmax, PImax, CPF, etc.)	92.3	NA	Highly recommended	
Hand Grip Strength	100	NA	Highly recommended	
Limb Muscle Strength (Manual Muscle Testing)	84.6	NA	Highly recommended	
MRC Sum Scale	87.2	NA	Highly recommended	
Skeletal Muscle Mass Index	84.6	NA	Recommended	
Borg Scale	100	NA	Highly recommended	
Visual Analog Scale	79.5	NA	Recommended	
Modified British Medical Research Council	100	NA	Highly recommended	
Target Dyspnea Ratings	79.5	NA	Recommended	
Barthel Index	74.4	NA	Recommended	
Functional Independence Measure	76.9	NA	Recommended	
Nottingham Extended ADL Scale	82.1	NA	Highly recommended	
Barthel Index Dyspnea	84.6	NA	Recommended	
SpO_2_ Monitoring During Daily Activities	100	NA	Highly recommended	
Short Physical Performance Battery (SPPB) ^a^	92.3	NA	Highly recommended	
Japanese Version of the Frailty Criteria	94.9	NA	Recommended	
Pedometer	97.4	NA	Highly recommended	
Activity Tracker	97.4	NA	Highly recommended	
Home-Based Life-Space Assessment	69.2	43.6	No consensus	Re-rated in Round 2
Six-Minute Walk Test	100	NA	Highly recommended	
Shuttle Walking Test	79.5	NA	Recommended	
Cardiopulmonary Exercise Testing	79.5	NA	Recommended	
Repetitive Saliva Swallowing Test	79.5	NA	Recommended	
Modified Water Swallow Test	71.8	NA	Recommended	
St. George's Respiratory Questionnaire	94.9	NA	Recommended	
Chronic Respiratory Disease Questionnaire	71.8	NA	Recommended	
Short-Form 36-Item Health Survey	79.5	NA	Recommended	
Japanese Version of EuroQol 5 Dimension	69.2	51.3	No consensus	Re-rated in Round 2
Hospital Anxiety and Depression Scale	92.3	NA	Recommended	
Patient Health Questionnaire-9	56.4	41.0	No consensus	Re-rated in Round 2
COPD Assessment Test	97.4	NA	Highly recommended	
**Domain 7. Able to Understand and Implement the Indications, Contraindications, and Evidence for the Following Programs:**				
Conditioning	100	NA	Highly recommended	
Endurance Training	100	NA	Highly recommended	
Muscle Strength Training	100	NA	Highly recommended	
ADL Training	100	NA	Highly recommended	
Respiratory Assistance and Cough Assistance	94.9	NA	Highly recommended	
Suctioning	100	NA	Highly recommended	

Abbreviations: ADLs, Activities of Daily Living; BMI, Body Mass Index; CO2, Carbon Dioxide; COPD, Chronic Obstructive Pulmonary Disease; CPF, Cough Peak Flow; CT, Computed Tomography; DLCO, Diffusing Capacity of the Lungs for Carbon Monoxide; FEV, Forced Expiratory Volume; MRC Sum Scale, Medical Research Council Sum Scale; MRI, Magnetic Resonance Imaging; PEmax, Maximum Expiratory Pressure; PETCO2, End-Tidal Carbon Dioxide Pressure; PImax, Maximum Inspiratory Pressure; SpO2, Peripheral Oxygen Saturation; %IBW, Percentage of Ideal Body Weight; %LBW, Percentage of Lean Body Weight; VC, Vital Capacity.

Domains/categories (for rapid scanning): Domain 1, knowledge of CRDs; Domain 2, medical devices/equipment; Domain 3, mechanical ventilation; Domain 4, pathophysiology; Domain 5, medical tests; Domain 6, assessment items; Domain 7, programs.

a The SPPB includes a standing balance subtest (side-by-side, semi-tandem, and tandem stance) as one component.

* No consensus items (did not reach ≥70% rating 4 or 5 after Round 2).

Note: Round 1 and Round 2 columns show the percentage of panelists rating each item as 4 (recommended) or 5 (highly recommended). Only items that did not meet the ≥ 70% inclusion criterion in Round 1 were re-rated in Round 2; items not re-rated in Round 2 are shown as “NA”. Final designation is based on the prespecified criteria (consensus for inclusion: ≥ 70% rating 4 or 5; Highly recommended: median = 5; Recommended: median = 4; No consensus: did not reach ≥70% after Round 2). Thus, items classified as “highly recommended” represent stronger consensus and can be identified within each domain/category.

In this table, ‘Noninvasive Ventilation’ is used as an umbrella term that includes bilevel positive airway pressure (often referred to clinically as BiPAP). Continuous Positive Airway Pressure is listed separately as a distinct candidate item. Dedicated balance impairment and falls-related constructs were not included as standalone candidate items in this Delphi and therefore were not evaluated as independent domains (see Limitations).

In summary, the items recommended as minimum standards spanned seven domains/categories of physical therapy practice (Table 2 and S3–S5 Tables). These included patient assessment (e.g., level of consciousness, physical assessment of the respiratory system), functional evaluation (e.g., muscle strength, ADL performance, exercise capacity), and patient-reported outcomes (e.g., dyspnea status, health-related QOL, and psychological status) (Table 2), which summarizes consensus ratings for the 114 prespecified candidate items. For terminology clarity within the mechanical ventilation domain, ‘Noninvasive Ventilation’ is used here as an umbrella term that includes bilevel positive airway pressure (BPAP; often referred to clinically as BiPAP), whereas Continuous Positive Airway Pressure is listed separately as a distinct candidate item.

## Discussion

This e-Delphi study established expert consensus among physical therapists on 105 minimum standards (knowledge and skills) considered necessary by the panel for physical therapists providing rehabilitation-oriented care for people with CRDs in Japan, including but not limited to PR programs. These standards may provide a foundation for harmonizing physical therapy practice and informing education and training in Japan.

The methodological rigor of this study is supported by its panel size and high participant retention. The panel size and completion rate were comparable to those reported in previous Delphi studies that developed minimum standards for physical therapists in specialized practice settings [34,36–38]. Retention across the two rounds was high (89%), which supports the stability of the consensus process and aligns with recommendations to report and consider attrition in Delphi studies [58]. The provision of anonymised group feedback between rounds, a common feature of (e-)Delphi designs, may also help sustain engagement in iterative consensus processes [48].

A key finding is that the standards identified in this study map closely onto domains emphasized in international initiatives to standardize outcome measurement in CRD rehabilitation care, including pulmonary rehabilitation [17,28,40]. Important domains such as exercise capacity, health-related QOL, and muscle function are consistent with the core domains prioritized in a recent core outcome set for pulmonary rehabilitation in COPD [40] and with work to define outcome domains and measures for COPD in physical therapy practice [17]. At the same time, international perspectives highlight ongoing heterogeneity in outcome measurement and assessment practices in pulmonary rehabilitation and related CRD rehabilitation care, underscoring the need for context-sensitive standardization [28,30].

Although the present study focused on physical therapists providing rehabilitation care for people with CRDs, our findings also show conceptual commonalities with minimum standards developed for physical therapists working in critical care settings, particularly with respect to core assessment competencies (e.g., respiratory assessment, muscle strength, functional mobility, activities of daily living, and cough effectiveness) [34,36–38]. These overlaps suggest that certain foundational knowledge and skills may be shared across clinical settings, while the required depth and context of application differ by setting. Importantly, we reference ICU minimum standards solely to contextualize areas of overlap and boundary conditions; this study does not aim to establish minimum standards for physical therapy in the ICU.

The lack of consensus on invasive line/monitoring–related items (e.g., arterial line and central venous catheter; all remaining below the prespecified 70% threshold across both rounds, Table 2) should be interpreted as delineating boundary conditions for minimum standards intended to apply across CRD rehabilitation settings. Because the Delphi survey did not elicit structured qualitative rationales for ratings, the following interpretation is necessarily contextual and inferential.

Notably, the panel endorsed several device-related competencies that may be encountered across CRD care pathways (e.g., oxygen delivery systems, noninvasive ventilation, tracheostomy-related devices, and suctioning), whereas vascular/invasive line items did not reach consensus. One plausible explanation is that invasive vascular monitoring is highly embedded in ICU workflows for critically ill patients and is closely coupled with safety management during mobilization, but expectations regarding the physical therapist’s role in interpreting or troubleshooting such devices may vary across institutions and multidisciplinary teams. Guidance on ICU mobilization safety describes device-related adverse events (including intravascular catheter removal or dysfunction) as key considerations during mobilization, underscoring that invasive devices sit within a high-acuity safety framework [60,61]. In contrast, in many routine CRD rehabilitation contexts (e.g., outpatient and community-based services), invasive vascular monitoring is not routinely available and is less commonly a prerequisite for physical therapy assessment. Therefore, non-endorsement of invasive line/monitoring–related items should not be interpreted as suggesting these practices are unimportant; rather, it indicates that they may be better positioned as setting-specific competencies contingent on patient acuity, resources, and local role delineation.

This study has several limitations. The first relates to the composition of the expert panel. As it consisted exclusively of physical therapists who were JSRPT members, the findings may have limited generalizability due to geographical and cultural specificity. In addition, because participation was voluntary, self-selection (volunteer) bias is possible. Accordingly, the panel should be interpreted as reflecting the perspectives of participating experts, and volunteer self-selection may have influenced which viewpoints were represented in the consensus process. Furthermore, the panel was composed primarily of physical therapists from medical and educational institutions, potentially excluding the perspectives of those working in care facilities. Moreover, because rehabilitation care for people with CRDs is multidisciplinary, the exclusive focus on physical therapists means that the perspectives of other relevant healthcare professionals (e.g., physicians, nurses, and occupational therapists) were not incorporated, which may limit broader acceptability and transferability across team-based care pathways. Accordingly, these findings should be interpreted as PT-specific minimum standards, rather than interprofessional minimum standards. Future research should engage multidisciplinary stakeholders to assess the acceptability, contextual relevance, and implementation considerations of these PT-specific standards within team-based CRD rehabilitation, and to inform future interprofessional consensus or adaptation work.

Second, limitations are inherent to the e-Delphi method. The consensus reflects current opinions that may evolve over time due to advancements in medical knowledge and shifts in expert perspectives; therefore, the identified minimum standards should be periodically updated. Additionally, the study relied solely on quantitative ratings to reach a consensus; we did not include open-ended questions or conduct qualitative evaluations, and therefore could not systematically examine panelists’ rationales for endorsing or not endorsing specific items, nor how individual panelists operationalized specific technical items. Accordingly, interpretations of why particular items did or did not reach consensus should be considered contextual inferences rather than empirically confirmed explanations.

Third, limitations relate to content scope and real-world applicability. Because the candidate item list was developed through a targeted review of selected documents (rather than a systematic/scoping review), some potentially relevant concepts may not have been included. Although the final minimum standards include the SPPB, which contains a standing balance subtest [62], balance impairment and falls-related constructs were not included as standalone candidate items and therefore were not evaluated as independent domains in the Delphi process. This is important because systematic reviews in COPD report clinically meaningful balance impairment compared with healthy controls and support consideration of balance assessment and training within pulmonary rehabilitation, and a more recent systematic review/meta-analysis indicates that exercise-based interventions can improve several balance outcomes while effects on falls remain uncertain and targeted balance training may provide the greatest benefits [63,64]. Future updates should therefore consider whether dedicated balance assessment and falls-related outcomes should be incorporated into an expanded candidate item list and evaluated in an updated consensus process. The Delphi-derived minimum standards reflect consensus on a prespecified candidate item list and are intended to define core knowledge and skills rather than a disease-specific protocol. Because CRDs encompass heterogeneous diagnoses and severities, some subtype- or stage-specific considerations may require local adaptation. Furthermore, entities not included in the prespecified candidate list (e.g., pulmonary edema and atelectasis) were not assessed and are therefore not reflected in the final set.

Finally, external validation is pending: the feasibility, acceptability, and practical operationalization of these standards (e.g., how they inform assessment selection and exercise intensity adjustment in routine settings) should be evaluated in future implementation studies.

## Conclusion

This e-Delphi study identified 105 consensus-based minimum-standard items (knowledge and skills) for physical therapists providing rehabilitation care for people with CRDs in Japan. These standards could inform training curricula and continuing professional development, could help guide policy-making and service planning by clarifying baseline expectations across care settings, and may provide a reference point for future cross-national comparisons following contextual adaptation. Future studies should evaluate implementation feasibility in routine practice and support periodic review and updating of the standards as clinical evidence and service contexts evolve.

## Supporting information

S1 Table Checklist of the CREDES guidelines for conducting and reporting Delphi studies. (DOCX)

S2 Table Item-level audit trail of pre-Delphi amendments between the archived draft item list and the final Round 1 e‑Delphi questionnaire items (n = 114). The table lists the final English item labels (as in Table 2), pre-Delphi amendment codes, one-line rationales, and informational source documents. PreDelphi_Amendment_Code reflects changes between the archived draft questionnaire and the final e-Delphi Round questionnaire used in the Delphi survey. ‘Source document(s)’ indicate informational inputs screened during item generation (targeted document review) and do not necessarily represent a single direct source for each item. Notes may include scope clarifications and mapping decisions made when operationalizing broad concepts from source documents into questionnaire items. Committee‑synthesized items were included where needed to reflect the Japanese clinical context.
(XLSX)

S1 Appendix Pre-Delphi item generation and refinement. (DOCX)

S3 Table Round-by-round agreement and descriptive statistics for each item (n = 114), organized by domain/category (Domains 1–7). Abbreviations: ADLs, Activities of Daily Living; BMI, Body Mass Index; CO2, Carbon Dioxide; COPD, Chronic Obstructive Pulmonary Disease; CPF, Cough Peak Flow; CT, Computed Tomography; DLCO, Diffusing Capacity of the Lungs for Carbon Monoxide; FEV, Forced Expiratory Volume; MRC Sum Scale, Medical Research Council Sum Scale; MRI, Magnetic Resonance Imaging; PEmax, Maximum Expiratory Pressure; PETCO2, End-Tidal Carbon Dioxide Pressure; PImax, Maximum Inspiratory Pressure; SpO2, Peripheral Oxygen Saturation; %IBW, Percentage of Ideal Body Weight; %LBW, Percentage of Lean Body Weight; VC, Vital Capacity. ^a^Items for which no consensus was reached. Note: Unindented rows indicate domain/category headings; indented rows list individual items. Round 1 and Round 2 columns show the percentage of panelists rating each item as 4 (recommended) or 5 (highly recommended). Only items that did not meet the ≥ 70% inclusion criterion in Round 1 were re-rated in Round 2; for all other items, the Round 2 column is shown as “NA”.
(XLSX)

S4 Table Final consensus designation (“highly recommended,” “recommended,” or “no consensus”) for all candidate items (n = 114), organized by domain/category. Abbreviations: ADLs, Activities of Daily Living; BMI, Body Mass Index; CO2, Carbon Dioxide; COPD, Chronic Obstructive Pulmonary Disease; CPF, Cough Peak Flow; CT, Computed Tomography; DLCO, Diffusing Capacity of the Lungs for Carbon Monoxide; FEV, Forced Expiratory Volume; MRC Sum Scale, Medical Research Council Sum Scale; MRI, Magnetic Resonance Imaging; PEmax, Maximum Expiratory Pressure; PETCO2, End-Tidal Carbon Dioxide Pressure; PImax, Maximum Inspiratory Pressure; SpO2, Peripheral Oxygen Saturation; %IBW, Percentage of Ideal Body Weight; %LBW, Percentage of Lean Body Weight; VC, Vital Capacity. Items designated as “highly recommended” or “recommended” (n = 105) constitute the final minimum standards.
(XLSX)

S5 Table Domain-by-consensus summary of final consensus designations for all candidate items (n = 114), organized by domain/category (Domains 1–7). (XLSX)

S1 Data Aggregated participant characteristic counts underlying Table 1 (n = 39). Counts and denominators are provided for each category; professional credentials allow multiple responses (percentages are calculated using n = 39 as the denominator; totals may exceed 100%).
(XLSX)

S2 Data Item-level denominators and category-level response counts by Delphi round used to calculate agreement rates and descriptive statistics. Rows represent item-by-round records. The 1–5 scale is: 1 = not at all recommended, 2 = not recommended, 3 = neutral, 4 = recommended, 5 = highly recommended. Agreement for consensus is defined as the proportion rated 4 or 5 (consensus threshold as specified in the Methods).
(XLSX)
