# Supplementary material for: Minimum standards for physical therapists providing rehabilitation care for people with chronic respiratory diseases in Japan: An e-Delphi study
Source: PLoS One. 2026 Mar 10;21(3):e0344464. doi: 10.1371/journal.pone.0344464 (PMC12974871; doi:10.1371/journal.pone.0344464)
Supplement: S1 Table — (DOCX) [file pone.0344464.s001.docx]

S1 Table. Checklist of the CREDES guidelines for conducting and reporting Delphi studies[1]

| **Recommendation** | Reported on page and line number |
| --- | --- |
| **Rationale for Delphi Technique** |  |
| ***Justification***. The choice of the Delphi technique as a method of systematically  collating expert consultation and building consensus needs to be well justified. | Page 7, lines 95–111. |
| **Planning and Design** |  |
| ***Planning and process***. The Delphi technique is a flexible method and can be adjusted to the respective research aims and purposes. Any modifications should be justified by a rationale and be applied systematically and rigorously | Page 7, lines 95–111; Pages 9–12, lines 161–221. |
| ***Definition of consensus***. Unless not reasonable due to the explorative nature of the study, an a priori criterion for consensus should be defined. This includes a clear and transparent guide for action on (a) how to proceed with certain items or topics in the next survey round, (b) the required threshold to terminate the Delphi process and (c) procedures to be followed when consensus is (not) reached after one or more iterations | Pages 11–12, lines 193–221. |
| **Study Conduct** |  |
| ***Informational input***. All material provided to the expert panel at the outset of the project and throughout the Delphi process should be carefully reviewed and piloted in advance in order to examine the effect on experts’ judgements and to prevent bias | Pages 9–12, lines 161–221. |
| ***Prevention of bias***. Researchers need to take measures to avoid directly or indirectly influencing the experts’ judgements. If one or more members of the research team have a conflict of interest, entrusting an independent researcher with the main coordination of the Delphi study is advisable | Pages 11–12, lines 192–221. |
| ***Interpretation and processing of results****.* Consensus does not necessarily imply the ‘correct’ answer or judgement; (non) consensus and stable disagreement provide informative insights and highlight differences in perspectives concerning the topic in question | Pages 34–36, lines 308–365. |
| ***External validation****.* It is recommended to have the final draft of the resulting guidance reviewed and approved by an external board or authority before publication and dissemination | Page 39, lines 416–416. |
| **Reporting** |  |
| ***Purpose and rationale****.* The purpose of the study should be clearly defined and demonstrate the appropriateness of the use of the Delphi technique as a method to achieve the research aim. A rationale for the choice of the Delphi technique as the most suitable method needs to be provided | Pages 6–7, lines 91–106. |
| ***Expert panel****.* Criteria for the selection of experts and transparent information on recruitment of the expert panel | Pages 7–9, lines 112–160. |
| ***Description of the methods****.* The methods employed need to be comprehensible; this includes information on preparatory steps, piloting of material and survey instruments, design of the survey instrument(s), the number and design of survey rounds, methods of data analysis, processing and synthesis of experts’ responses to inform the subsequent survey round and methodological decisions taken by the research team throughout the process | Pages 7–12, lines 95–221;  S1 Appendix. |
| ***Procedure****.* Flow chart to illustrate the stages of the Delphi process, including a preparatory phase, the actual ‘Delphi rounds’, interim steps of data processing and analysis, and concluding steps | Fig 1. |
| ***Definition and attainment of consensus****.* It needs to be comprehensible to the reader how consensus was achieved throughout the process, including strategies to deal with non-consensus | Pages 11–12, lines 192–221. |
| ***Results****.* Reporting of results for each round separately is highly advisable in order to make the evolving of consensus over the rounds transparent. This includes figures showing the average group response, changes between rounds, as well as any modifications of the survey instrument such as deletion, addition or modification of survey items based on previous rounds | Pages 14–16, lines 243–276; Table 2; S3–5 Table. |
| ***Discussion of limitations****.* Reporting should include a critical reflection of potential limitations and their impact of the resulting guidance | Pages 36–39, lines 366–419. |
| ***Adequacy of conclusions****.* The conclusions should adequately reflect the outcomes of the Delphi study with a view to the scope and applicability of the resulting practice guidance | Page 39, lines 422–430. |
| ***Publication and dissemination****.* The resulting guidance should be clearly identifiable from the publication, including recommendations for transfer into practice and implementation. A dissemination plan should include endorsement of the guidance by professional associations and health care authorities to facilitate implementation | Page 39, lines 428–430. |

**Reference**

1. Jünger S, Payne SA, Brine J, Radbruch L, Brearley SG. Guidance on Conducting and REporting DElphi Studies (CREDES) in palliative care: Recommendations based on a methodological systematic review. Palliat Med. 2017;31: 684–706.
