# Supplementary material for: Minimum standards for physical therapists providing rehabilitation care for people with chronic respiratory diseases in Japan: An e-Delphi study
Source: PLoS One. 2026 Mar 10;21(3):e0344464. doi: 10.1371/journal.pone.0344464 (PMC12974871; doi:10.1371/journal.pone.0344464)
Supplement: S1 Appendix — (DOCX) [file pone.0344464.s003.docx]

**S1 Appendix.** **Pre-Delphi item generation and refinement**

**A. Objective and scope**

This appendix documents the development of the candidate minimum standards questionnaire used in Delphi Round 1. The preparatory phase aimed to translate key concepts relevant to respiratory physical therapy practice in Japan into clear, answerable questionnaire items in Japanese and to standardize item wording prior to Delphi administration.

Items were considered out of scope if they were not directly applicable to respiratory physical therapy for people with chronic respiratory diseases (CRDs) across care settings. Therefore, when screening Takahashi et al. (Intensive Care Unit (ICU) minimum standards), we excluded highly ICU-specific devices/monitoring and non-respiratory critical-care conditions (e.g., IMPELLA or other extracorporeal circulation devices, Bispectral Index (BIS) monitoring, hemorrhagic shock, sepsis), retaining only respiratory-related acute-care concepts (e.g., oxygen therapy, ventilatory support, and acute exacerbation management).

**B. Information sources**

Candidate concepts were developed through a targeted review of key documents selected a priori by the committee to ensure coverage of essential domains and relevance to the Japanese clinical context (this preparatory phase was a targeted document review rather than a systematic/scoping review). Primary Japan-specific sources included (i) the national clinical practice guideline for physical therapy in respiratory disorders [54], (ii) a Japanese review summarizing outcome measures used in respiratory physical therapy (to inform assessment/outcome concepts) [55], and (iii) a national Japanese statement on respiratory rehabilitation [56]. To incorporate an international perspective on pulmonary rehabilitation content and outcome domains, we also consulted an official American Thoracic Society workshop report defining modern pulmonary rehabilitation [18]. In addition, to cross-check coverage of patient education/self-management domains potentially applicable across CRDs, we screened a consensus paper identifying core pulmonary rehabilitation education topics in interstitial lung disease [41]; it was used for domain coverage checks rather than as a direct item source. Finally, to inform respiratory-related acute-care concepts that may be encountered across care pathways, we consulted a Japanese minimum-standards/Delphi study for physical therapists working in intensive care [38]; ICU-specific devices/monitoring and non-respiratory critical-care conditions were treated as out of scope (see Section A: Objective and scope).

Table A provides the key documents screened/consulted during the targeted pre-Delphi phase and their intended roles.

**Table A. Key documents screened/consulted during the targeted pre-Delphi phase and their intended roles**

| **Document (author/year)** | **Type** | **Primary domains informed** | **Reason for inclusion** |
| --- | --- | --- | --- |
| National guideline, 2021 (Japan) [54] | Clinical practice guideline | Knowledge domains; disease/pathophysiology; tests; interventions | Provided the primary national reference for respiratory physical therapy practice in Japan. |
| Sekikawa et al., 2022 [55] | Review (outcome measures; Japan) | Outcome measures; assessment concepts | Provided a Japan-specific compilation of commonly used/validated outcome measures in respiratory physical therapy. |
| National statement, 2018 (Japan) [56] | Professional statement | Assessment domains; program concepts; education topics | Provided consensus-based domains and priorities for respiratory rehabilitation in Japan. |
| American Thoracic Society statement, 2021 [18] | Definition/consensus (pulmonary rehabilitation) | Program components; delivery; selected assessment and education concepts | Provided internationally accepted components of pulmonary rehabilitation and related assessment/education concepts. |
| Holland et al., 2019 [41] | Consensus ( interstitial lung disease (ILD) pulmonary rehabilitation education topics) | Education/self-management topics | Screened to cross-check coverage of patient education/self-management domains; ILD-specific topics were screened for scope and the document was not used as a direct item source. |
| Takahashi et al., 2021 [38] | Minimum standards (Delphi; ICU physical therapy, Japan) | Devices/equipment; ventilation; monitoring; selected acute-care concepts | Consulted as a methodological exemplar and to inform respiratory-related acute-care concepts encountered across care pathways; the present study did not aim to develop ICU-specific standards. ICU-specific devices/monitoring and non-respiratory critical-care conditions were treated as out of scope (see Section A: Objective and scope). |

**C. Item extraction and drafting (initial extraction by three committee members)**

Three physical therapists from the Japanese Society of Respiratory Physical Therapy (JSRPT) Standardization Committee extracted candidate concepts from the above sources and converted them into draft questionnaire items. During drafting, the committee aimed to express one concept per item and to use Japanese clinical terminology commonly used by the target population.

**D. Pre-Delphi committee review and refinement (review by three additional committee members)**

The preliminary item pool was reviewed by three additional physical therapists from the JSRPT Standardization Committee. A set of consistent refinement rules was applied to improve clarity and reduce redundancy, including: (i) removal of duplicates, (ii) merging overlapping concepts, (iii) splitting double-barrelled items, (iv) wording clarification and terminology harmonization, and (v) scope specification when needed to avoid overly broad or overly narrow interpretation.

**E. Pretest of the draft online questionnaire**

Prior to Delphi Round 1, the draft questionnaire was pretested by six committee members to confirm comprehensibility and feasibility, including Google Forms functionality. Minor wording revisions were made during the pre-Delphi refinement process to improve clarity and consistency. A separate external pilot with non-panelists was not conducted.

**F. Output of the preparatory phase**

Following the above steps, the Round 1 questionnaire comprised 114 items and was administered in Japanese using Google Forms. An item-level audit trail of pre-Delphi amendments is provided in S2 Table. For readability, per-item ‘Source document(s)’ listings in S2 Table were limited to the most relevant informational inputs; the full set of screened documents and their intended roles are summarized in Table A.

**G. Representative examples of item refinement (before/after)**

Table B summarizes the representative examples of how extracted concepts were refined into the final Round 1 items prior to the Delphi rounds.

**Table B. Representative examples of how extracted concepts were refined into the final Round 1 items prior to the Delphi rounds (before/after).**

| **Example ID** | **Archived draft wording (Japanese)** | **Refinement action** | **Final Round 1 wording (Japanese)** | **Rationale** |
| --- | --- | --- | --- | --- |
| Ex-1 | 慢性呼吸不全患者に対する以下の知識を有している (Have knowledge of the following for people with chronic respiratory failure) | Terminology harmonization / wording clarification (TERM; REWORD) | 慢性呼吸器疾患に対する以下の知識を有している (Have knowledge of the following regarding chronic respiratory diseases) | Harmonize terminology with the target population (chronic respiratory diseases) and standardize wording across items. |
| Ex-2 | 酸素療法：在宅酸素療法（酸素濃縮器） (Oxygen therapy: Home oxygen therapy (oxygen concentrator)) | Scope specification / wording clarification (SCOPE; REWORD) | 酸素療法：在宅酸素療法 (Oxygen therapy: Home oxygen therapy) | Avoid device-specific wording to improve applicability across clinical settings and devices. |
| Ex-3 | 咳嗽能力の評価 (Assessment of cough ability) | Operational definition added (DEFN; REWORD) | 咳嗽能力の評価 (cough peak flow) (Assessment of cough ability (cough peak flow)) | Reduce ambiguity by specifying an interpretable/operational measure. |
| Ex-4 | (Not present in archived draft list) | Item added (ADD) | 慢性呼吸器疾患に対する以下の知識を有している [多職種介入・呼吸ケアサポートチーム] (Have knowledge of the following regarding chronic respiratory diseases [Multidisciplinary interventions and respiratory care support teams]) | Added during pre-Delphi refinement to address a potentially missing concept and to improve completeness of minimum standards. |

Note: Japanese wording is shown; English translations are provided in parentheses. The item-level audit trail (S2 Table) was compiled based on the archived draft document and the final Round 1 questionnaire used in the Delphi survey.
